# Poly(lactic-co-glycolic) Acid-Chitosan Dual Loaded Nanoparticles for Antiretroviral Nanoformulations

**DOI:** 10.1155/2016/3810175

**Published:** 2016-04-17

**Authors:** Faithful Makita-Chingombe, Hilliard L. Kutscher, Sara L. DiTursi, Gene D. Morse, Charles C. Maponga

**Affiliations:** ^1^School of Pharmacy, University of Zimbabwe, P.O. Box MP167, Mount Pleasant, Harare, Zimbabwe; ^2^The Institute of Lasers, Photonics and Biophotonics, University at Buffalo, Buffalo, NY 14260, USA; ^3^Center for Integrated Global Health Sciences, Translational Pharmacology Research Core, New York State Center of Excellence in Bioinformatics and Life Sciences, University at Buffalo, Buffalo, NY 14203, USA; ^4^School of Pharmacy and Pharmaceutical Sciences, University at Buffalo, Buffalo, NY 14214, USA

## Abstract

Poly(lactic-co-glycolic acid) (PLGA) chitosan (CS) coated nanoparticles (NPs) were loaded with two antiretrovirals (ARVs) either lamivudine (LMV) which is hydrophilic or nevirapine (NVP) which is hydrophobic or both LMV and NVP. These ARVs are of importance in resource-limited settings, where they are commonly used in human immunodeficiency virus (HIV-1) treatment due to affordability and accessibility. NPs prepared by a water-oil-water emulsion and reduced pressure solvent evaporation technique were determined to have a positive zeta potential, a capsule-like morphology, and an average hydrodynamic diameter of 240 nm. Entrapment of NVP as a single ARV had a notable increase in NP size compared to LMV alone or in combination with LMV. NPs stored at room temperature in distilled water maintained size, polydispersity (PDI), and zeta potential for one year. No changes in size, PDI, and zeta potential were observed for NPs in 10% sucrose in lyophilized or nonlyophilized states stored at 4°C and −20°C, respectively. Freezing NPs in the absence of sucrose increased NP size. Drug loading, encapsulation efficiency, and kinetic release profiles were quantified by high performance liquid chromatography (HPLC). Our novel nanoformulations have the potential to improve patient outcomes and expand drug access in resource-limited countries for the treatment of HIV-1.

## 1. Introduction

HIV infection is one of the deadliest diseases worldwide, particularly in resource-limited settings. Antiretrovirals (ARVs) have offered life-sustaining treatment for people living with HIV infection and acquired immunodeficiency syndrome (AIDS). As of March 2015, ARV access remains limited with only 15 million (~40%) of the 36.9 million individuals infected worldwide receiving treatment [1, 2]. Combination antiretroviral therapy (cART) is highly effective because it targets multiple stages of the HIV lifecycle. While patient access to ARVs is increasing, adverse effects, large pill burden, and frequent administration of many first generation ARVs have led to their reduced use. This provides an opportunity to reformulate currently approved cART therapies.

The World Health Organization (WHO) guidelines for the use of ARVs in treating HIV infection in adults recommend NVP in combination with zidovudine/lamivudine or tenofovir/lamivudine when combination therapy with tenofovir/lamivudine/efavirenz is contraindicated or not available [3]. NVP and LMV are still utilized as first-line ARVs particularly in resource-limited settings due to their availability and lower cost. Although more efficacious regimens have been recommended by the WHO, their availability has been largely delayed in resource-limited settings. Additional barriers also exist that make adherence to the prescribed regimen a challenge for patients being treated for HIV such as stigma, complexity of regimens, pill burden/fatigue, food requirements, adverse effects, nondisclosure, failure to fill prescriptions, and cost-related issues [4]. Therefore, long-acting, cell-targeted ARVs have become a major area of research.

NPs have revolutionized sustained drug delivery and cell specific targeting approaches and have been developed to deliver conventional drugs, recombinant proteins, vaccines, and nucleotides. Modifying plasma exposure through sustained release profiles and/or cell targeting can further reduce the toxicities associated with these therapeutics. Therapeutic agents that are efficacious but have serious adverse effects and/or toxicities have the potential to be reinvestigated and reformulated as NPs in order to diminish or eliminate these unfavorable properties. For example, doxorubicin is an FDA approved chemotherapeutic agent that was reformulated into PEGylated liposomes (Doxil®). Doxil demonstrated enhanced antitumor efficacy compared to doxorubicin alone and had a lower incidence of toxicities, most notably cardiotoxicity [5]. Paclitaxel, an FDA approved chemotherapeutic, was reformulated into albumin-based NPs (Abraxane®). Abraxane greatly reduced adverse effects associated with the former formulation [6].

A major concern with reformulation of a drug is maintaining its stability during manufacturing and storage. Biodegradable and biocompatible polymers such as poly(lactic-co-glycolic acid) (PLGA) have been shown to protect drug molecules from enzymatic degradation and provide physicochemical stability [7].

NPs can be optimized by size and shape or functionalized with protein and lipid coatings to facilitate their drug release, cellular uptake, and ability to cross physiologic barriers, for example, the blood-brain barrier [8–10]. Furthermore, NPs can be functionalized with ligands such as those with immune system modulating effects, concurrently modifying the cellular immune response and enhancing intracellular drug delivery [11]. Chitosan (CS) has gained attention in the nanomedicine field because it carries a positive charge that can be utilized for cellular and anatomic targeting of NPs [12]. The electrostatic interactions between positively charged CS NPs and the negatively charged cell surface have been shown to enhance nanoparticle uptake [13–18]. By using a PLGA core in conjunction with a CS shell, both hydrophobic and hydrophilic drugs can be encapsulated within NP.

The objective of this study was to prepare and characterize NVP- and LMV-NPs both as single ARVs and in combination and to assess their physicochemical stability.

## 2. Materials and Methods

### 2.1. Materials

Poly(lactic-co-glycolic acid) (PLGA) (lactide : glycolide = 50 : 50, molecular weight (MW) = 38–54 kDa), poly(vinyl alcohol) (PVA) (Mowiol® 4-88, MW = 31 kDa), and chitosan (CS) oligosaccharide lactate (MW = 5 kDa) were purchased from Sigma Aldrich (St. Louis, MO). Nevirapine (NVP) and lamivudine (LMV) were purchased from TCI America (Portland, OR). All other reagents were used as received without further purification.

### 2.2. Preparation of NPs

NPs were prepared using a modified water-oil-water (w/o/w) double emulsion and solvent evaporation technique [19]. LMV, NVP, or both (4 mg) were added to a microcentrifuge tube containing 200 *μ*L water with 90 *μ*g of CS and 500 *μ*L of 10 mg/mL PLGA dissolved in dichloromethane (DCM). The microcentrifuge tube was probe sonicated (750-watt probe sonicator, 1/4-inch probe tip, Cole-Parmer Vernon Hills, IL) at 21% amplitude for 30 seconds in a 4°C water bath. The resulting emulsion was quickly added to 2 mL of 2.5% (w/v) PVA in water in a 5 mL centrifuge tube and probe sonicated again for 90 seconds in a 4°C water bath to obtain the w/o/w emulsion. The resulting w/o/w emulsion was quickly added dropwise to 10.25 mL of 0.025% PVA in a 100 mL round bottom flask with a stir bar. Under constant stirring, DCM was evaporated under vacuum. The NP suspension was washed once with water followed by centrifugation at 14,000 ×g for 15 minutes. NPs were resuspended in water or 10% (w/v) sucrose prior to storage or characterization.

### 2.3. Characterization of NPs

NP size and polydispersity index (PDI) were determined by 3 ways: (1) dynamic light scattering (DLS) (Brookhaven 90 plus Particle Analyzer, Brookhaven Instruments, Holtsville, NY) at an angle of 90°; (2) nanoparticle tracking analysis (NTA) (LM-10, Malvern Instruments, Westborough, MA); and (3) transmission electron microscopy (TEM). Surface morphology was analyzed by TEM. TEM samples were prepared by staining with 0.1% w/v phosphotungstic acid. Zeta potential was determined by Zeta PALS (Brookhaven Instruments). ARV loading and* in vitro* release were characterized by high performance liquid chromatography (HPLC) (Alliance 2795, Waters Corporation, Milford, MA).

### 2.4. Chromatographic Separation

Detection of NVP and LMV was adapted from Rezk et al. [20]. ARVs were separated by reverse phase chromatography on an Atlantis dC_18_ column (3.9 × 150 mm, 5 *μ*m, Waters Corporation) with an Atlantis dC_18_ guard column (3.9 × 20 mm, 5 *μ*m, Waters Corporation) and detected using a photodiode array detector (PDA 996, Waters Corporation) collecting at 270 nm for LMV and 282 nm for NVP. Zalcitabine (ddC) was used as the internal standard (IS) and detected at both 270 and 282 nm. Chromatographic separation was performed with gradient elution. LMV, NVP, and IS eluted at 6.1, 14.1, and 7.7 minutes, respectively. The two mobile phase components were as follows: mobile phase A: 10 mM ammonium acetate; mobile phase B: 200 mL mobile phase A, 500 mL acetonitrile, and 300 mL methanol. Both mobile phases were filtered through a 0.22 *μ*m membrane filter (Millipore, Milford, MA). A linear gradient was programmed in which the initial percentages of mobile phases A and B were 96% and 4%, respectively. Mobile phase B changed to 10% at 8 minutes, 30% at 9.5 minutes, 43% at 13 minutes, and then to the initial 4% at 16.5 minutes up to 20 minutes. The mobile phase flow rate was 1 mL/minute, column temperature was set to 40°C, and injection volume was 100 *μ*L. Data were collected and processed using Waters Empower Chromatography Software. The calibration curve was linear from 200 ng/mL to 10,000 ng/mL for NVP and 50 ng/mL to 10,000 ng/mL for LMV.

### 2.5. ARV Loading and Encapsulation Efficiency

The amounts of NVP and LMV entrapped in the nanoparticles were determined using the HPLC method described above. A 1 mL aliquot of PLGA-CS NPs was centrifuged for 15 minutes at 14,000 ×g. The supernatant was removed and 0.5 mL acetonitrile and 0.5 mL water were added to dissolve the PLGA-CS NPs. Each sample was bath sonicated for 5 minutes followed by drying under nitrogen gas (Zymark TurboVap LV, Hopkinton, MA). Samples were then reconstituted with HPLC grade water before measuring ARV content by the HPLC method described above. The percentage of drug loading and encapsulation efficiency were calculated using the following equations:

(1) Drug loading efficiency=amount of drug loadedamount of PLGA×100%,Encapsulation efficiency=amount of drug loadedtotal drug amount×100%.

### 2.6. ARV Release Kinetics

Release kinetics of dual and singly loaded ARV PLGA-CS NPs were evaluated by taking 1 mL aliquots of PLGA-CS NPs. Each aliquot was washed twice with distilled water by centrifuging the sample for 15 minutes at 14,000 ×g and removing the supernatant. The pellet was resuspended in 1 mL of 10 mM phosphate buffer, pH 7.2 or pH 5, and added to a microcentrifuge tube. Microcentrifuge tubes were stored at 37°C or 4°C. At predetermined time points, a microcentrifuge tube was removed from the storage condition and centrifuged for 15 minutes at 14,000 ×g. From the microcentrifuge tube, 250 *μ*L of supernatant was aliquoted, spiked with IS, and analyzed for ARV using the HPLC method described above. Data were fit using GraphPad Prism 6.0 (La Jolla, CA) to several drug release models including zero order, first order, Higuchi, Korsmeyer-Peppas, and Hixson-Crowell to determine the release mechanism.

### 2.7. Stability of NPs

Size, PDI, surface morphology, surface charge (zeta potential), and drug encapsulation were determined at time of fabrication, two weeks, one month, and one year for single ARV or dual ARV-loaded NPs stored at room temperature, 4°C, −20°C, and −70°C. The stability of NPs lyophilized with and without 10% sucrose was also evaluated.

## 3. Results and Discussion

Recently, a paradigm shift in HIV research from drug discovery to drug delivery has occurred [21]. Novel ARV nanoformulations have shown improvements in pharmacokinetics which have the potential to simplify dosing strategies, increase patient adherence, and maximize antiviral efficacy [22, 23]. Rilpivirine (TMC-278, Edurant®) and cabotegravir (GSK1265744) are two ARVs currently in phase II/III trials as once monthly, long-acting injectables. Clinical trials have shown promise for these agents as long-acting NP injections for the treatment of HIV-1 infection. These novel formulations have been shown to maintain plasma concentrations significantly above the target plasma inhibitory concentration, reducing the likelihood of subtherapeutic plasma concentrations and thus reducing the potential for viral resistance. These formulations are administered monthly or even quarterly as opposed to once daily dosing, which can greatly improve patient adherence to ARVs.

We used an existing encapsulation method to load ARVs into polymeric NPs. The emulsion-solvent-evaporation technique is a proven method for encapsulating hydrophobic therapeutics but has poor results in encapsulating hydrophilic therapeutics. This study utilized a modification of this technique by adding an additional emulsification step which provides an opportunity to encapsulate hydrophilic agents [24, 25]. By fabricating NP with both PLGA and CS, we are able to successfully incorporate two ARVs that have markedly different aqueous solubilities and hydrophobicities (NVP: 0.1 mg/mL, log⁡*P* = 2.5; LMV: 70 mg/mL, log⁡*P* = −1.4).

### 3.1. Morphology and Size of Single and Dual Loaded NPs

**Figure 1:**
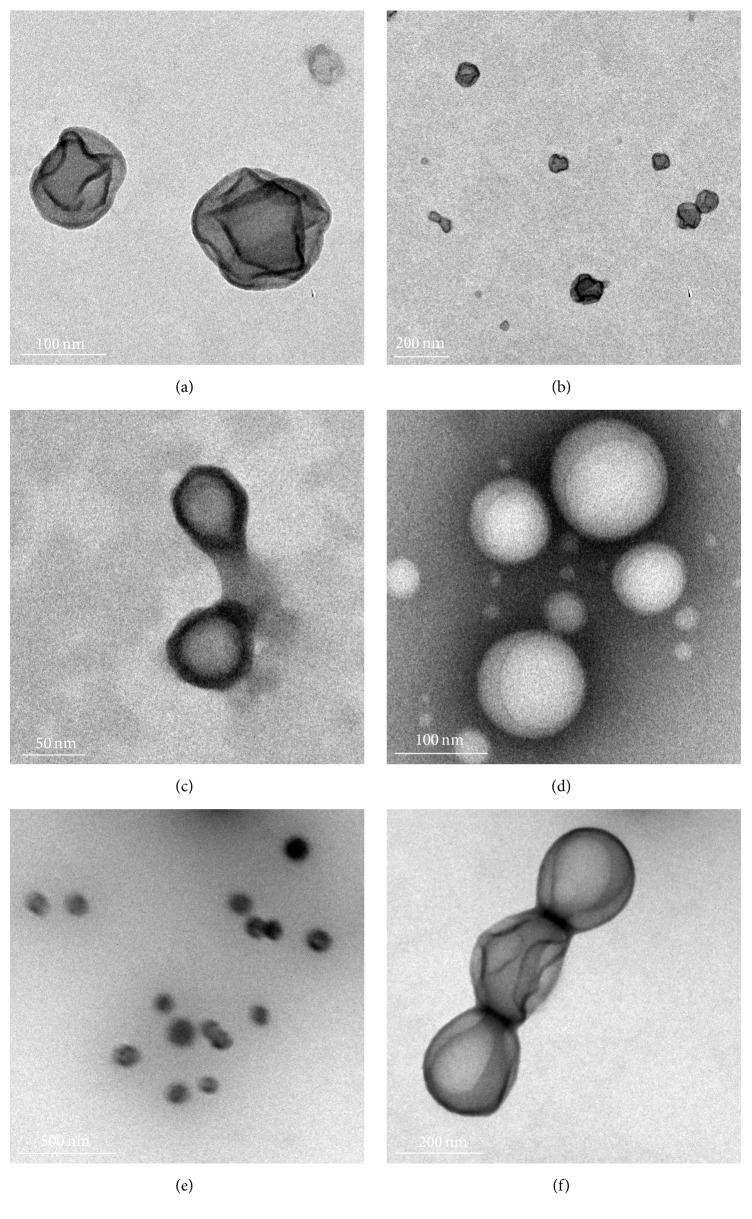
TEM image of PLGA-CS-NVP-LMV NPs. NPs following fabrication (a, b); after 1 year, stored at room temperature (c); after 1 year, stored at −70°C (d); lyophilized with 10% sucrose, stored at 4°C (e); and stored at −20°C in 10% sucrose but nonlyophilized (f).

Figure 1 illustrates the capsule morphology of ARV-loaded NPs confirming the existence of an inner core and an outer shell. We postulate that NVP is encapsulated within the hydrophobic inner core of PLGA while LMV is encapsulated within the hydrophilic CS outer shell.

**Figure 2:**
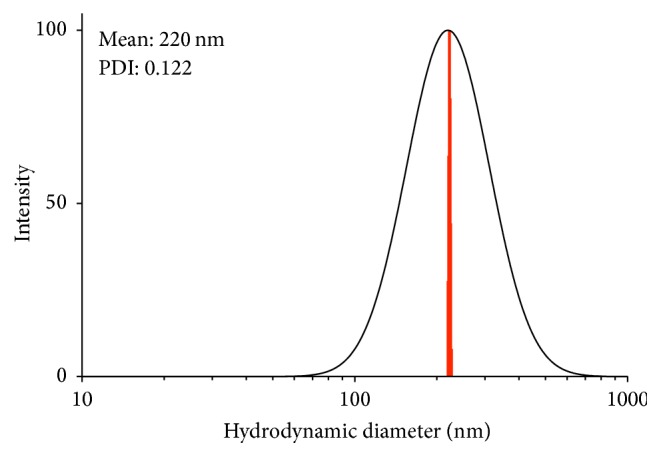
Log-normal distribution for PLGA-CS-NVP-LMV NPs (Sample 3.4). MSD histogram in red.

Small-sized NPs are associated with higher uptake by cells, offering an efficient delivery system. However, drug loading efficiency of passively entrapped therapeutics decreases with particle size [16]. To enable sufficient drug loading, we targeted NPs that are 250 nm in diameter. In this study, NPs had an average diameter of 242 nm (Table 1) measured by DLS. In DLS, the intensity weighted function of the hydrodynamic diameter is biased to larger NPs [26]. However, when comparing the number-weighted DLS result to NTA and TEM, they are comparable (Table 2). The low polydispersity and size distribution of Sample 3.4 is shown in Figure 2.

NP size was influenced by the hydrophobicity of the encapsulated ARV. PLGA-CS-LMV NPs had an average diameter of 237 nm, whereas PLGA-CS-NVP NPs had an average diameter of 282 nm. The increase in size of the NVP-loaded NPs can be attributed to encapsulation of the hydrophobic drug entrapped in the core leading to changes in the packing structure of the polymer upon NP fabrication. In addition, the physicochemical interactions between PLGA and NVP influenced the increased PDI of the PLGA-CS-NVP NPs compared to PLGA-CS-LMV NPs (Table 1). However, PDI remained below 0.2, which is considered a monodisperse system [27]. Monodisperse NPs are ideal in drug delivery as they tend to yield more consistent pharmacokinetic properties as a function of their size, for example, release kinetics and biodistribution [28, 29].

NPs formulated with CS exhibited a positive zeta potential suggesting successful coating with CS (Table 1). This overall positive charge is a direct result of the electrostatic interactions between the negatively charged PLGA and positively charged CS, indicating adsorption of CS onto the surface of the PLGA NP [11]. PLGA NPs formulated without CS (Table 1, Samples 5.1 and 6.1) were negatively charged, as consistent with the literature [15].

### 3.2. Stability Evaluation

#### 3.2.1. Stability of Prepared Single and Dual Loaded NPs

**Figure 3:**
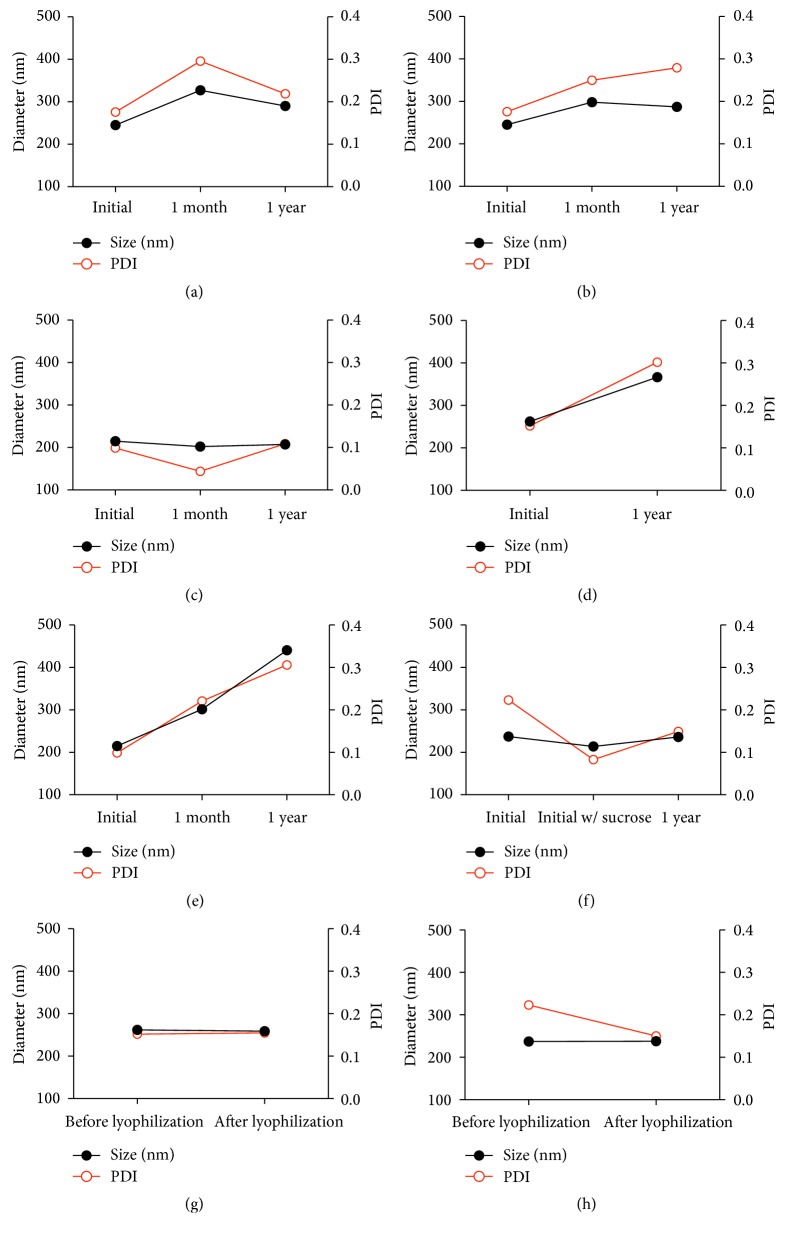
Size and PDI stability of NPs at different storage conditions; (a), (b), (c), and (f) were stable while (d) and (e) were unstable. Size is maintained after lyophilization (g and h). (a) PLGA-CS-NVP-LMV NPs stored at room temperature; (b) PLGA-CS-NVP NPs stored at room temperature; (c) PLGA-CS-LMV NPs lyophilized with 10% sucrose and stored at 4°C; (d) PLGA-CS-NVP-LMV NPs lyophilized without sucrose and stored at 4°C; (e) PLGA-CS-LMV NPs lyophilized without sucrose and stored at 4°C; (f) PLGA-CS-NVP NPs in 10% sucrose stored at −20°C; (g) PLGA-CS-NVP-LMV NPs lyophilized in 10% sucrose; (h) PLGA-CS-NVP NPs lyophilized in 10% sucrose.

A major area of concern for NPs is their long-term physical and chemical stability [30]. Long-term stability is generally defined to be at least 12 months [31]. To advance an ARV NP formulation towards clinical usage, the NP and incorporated ARV must be stable. Furthermore, given the limited resources available in developing countries, storage at room temperature is highly desirable. In this study, dual loaded NPs stored at room temperature showed little to no change in morphology (Figure 1(c)), size, PDI (Figure 3(a)), or zeta potential after one year. This stability was also observed for both PLGA-CS-NVP NPs (Figure 3(b)) and PLGA-CS-LMV NPs (data not shown).

This stability of NPs may be due in part to using PVA as a surfactant. Stabilizers such as PVA have been shown to enhance short-term and long-term stability of NP formulations [32]. PVA can act as a stabilizer, providing sufficient steric hindrance between NPs, allowing them to overcome colloidal instabilities. Moreover, PVA may act as a stabilizer by adsorbing to the NP surface, forming an outer layer that is not easily removed during washing.

In this study, NPs stored at −70°C for one year were larger in size with a mean of 500 nm using DLS and 200 nm using TEM (Figure 1(e)). However, PDI and zeta potential showed no marked differences upon storage at −70°C for one year (not shown). Between −70°C and 0°C, freezing and nonfreezing water molecules bind to polymer molecules through hydrogen bonds [33]. Hydrogen bond interactions between the OH and NH_2_ groups of chitosan and the OH groups of PVA may cause the NPs to aggregate, resulting in an apparent increase in NP size [34].

#### 3.2.2. Stability of Lyophilized NPs

Lyophilization has been shown to enhance long-term stability of colloidal NPs [30]. It has been reported that NPs prepared with 2.5–5% PVA may be lyophilized without the use of a cryoprotectant [30]. However, following washing to remove excess ARV, CS, and PVA, the addition of a cryoprotectant such as sucrose may be required to ensure stability. NPs lyophilized with 10% sucrose readily dispersed in water upon reconstitution both immediately after lyophilization and following storage at 4°C for one year. The presence of an easily dispersible lyophilized powder and conservation of NP size (Figures 3(g) and 3(h)) indicate successful lyophilization of the NPs [30]. At one year, the capsule morphology (Figure 1(e)), size, PDI (Figure 3(c)), and surface charge of NPs lyophilized with sucrose were preserved. These results are similar to changes in size when PLGA NPs lyophilized with 1–3% sucrose were stored at 4°C and 25°C for 3 months [35]. However, the size and PDI of dual loaded NPs lyophilized in the absence of sucrose increased when stored at 4°C for one year (Figure 3(d)). This change in size and PDI was also observed for single loaded PLGA-CS-LMV NPs lyophilized in the absence of sucrose (Figure 3(e)). These results demonstrate the importance of using sucrose as a cryoprotectant. Finally, nonlyophilized NPs in 10% sucrose stored at −20°C for one year had no notable differences in morphology (Figure 1(f)), size, or PDI (Figure 3(f)) when compared to NPs directly following their fabrication.

### 3.3. *In Vitro* ARV Release Kinetics and Mechanism

**Figure 4:**
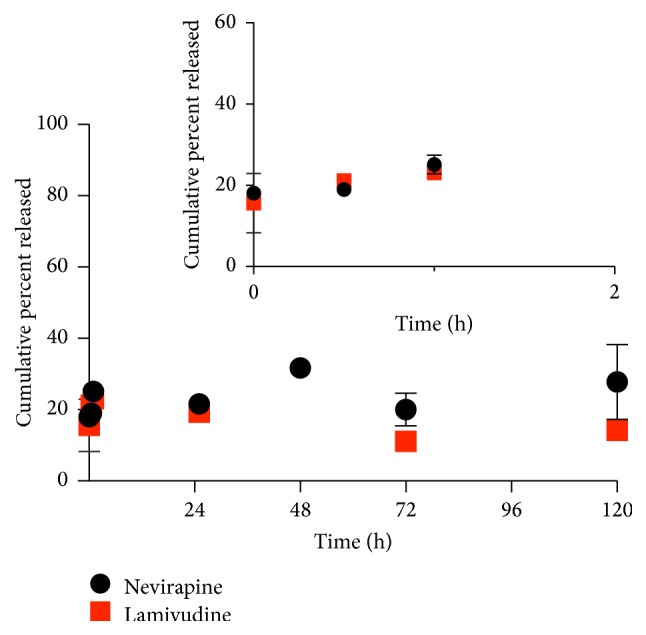
Release of LMV and NVP from PLGA-CS-NVP-LMV NPs at 37°C in PBS pH 7.2 (mean *n* = 3).

Prolonged release of ARVs from polymeric NPs has been reported [36]. However,* in vivo* ARV release and drug pharmacokinetics may differ from* in vitro* results [37]. In particular, lysosomes and proteasomes play an important role in the destruction of HIV-1 during endocytosis of the virus. Apart from HIV being internalized by fusion to the cell membrane, HIV can also enter the cell via endocytosis if spared from degradation in late endosomes and lysosomes [38]. If the HIV pathogen remains intact and viable in the endosome, the cell becomes prone to viral infection [39]. Although the internalization of HIV by endocytosis does not result in productive infection [38], it has been reported that infectivity of HIV-1 is enhanced by the inhibition of lysosomal function [39]. In order to predict future biological efficacy, this study sought to determine the drug release kinetics and possible mechanism of LMV and NVP from single or dual loaded NPs. Release studies were performed in phosphate buffer at 37°C at pH 7.2 and pH 5 in order to mimic physiologic and late stage lysosomal pH, respectively [40]. At 37°C in pH 7.2 (Figure 4), both LMV and NVP in the dual loaded NPs exhibited a biphasic release profile. An initial burst in the first 20 hours was observed for LMV followed by a slow and almost stable release over the remaining 100 hours. Concurrently, within the first 20 hours, NVP was slowly released initially but gradually surpassed LMV levels over the remaining 100 hours.

**Figure 5:**
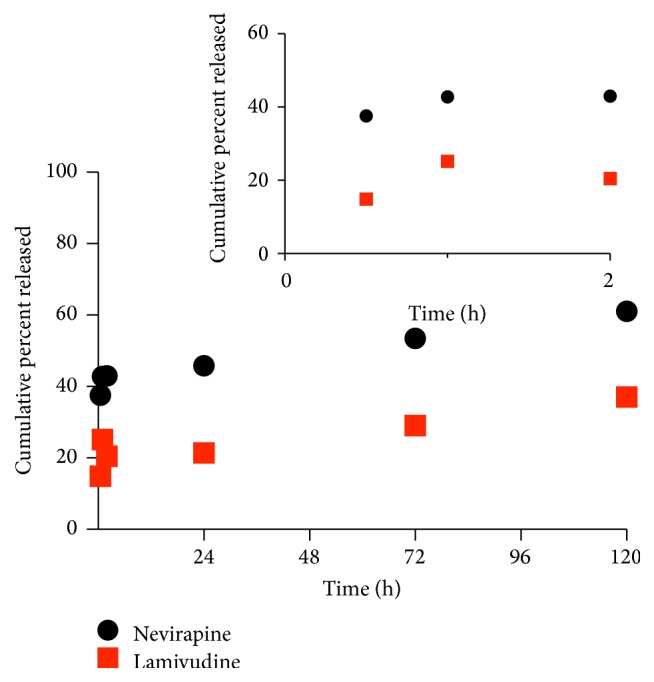
Release of LMV and NVP from PLGA-CS-NVP-LMV NPs at 4°C in PBS pH 7.2 (mean *n* = 3).

**Figure 6:**
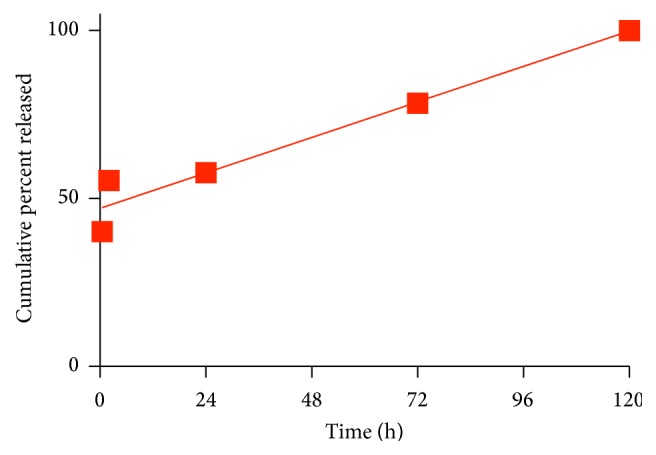
Release kinetics of PLGA-CS-LMV NPs in pH 5 at 37°C. Data are fit using the zero-order model.

**Figure 7:**
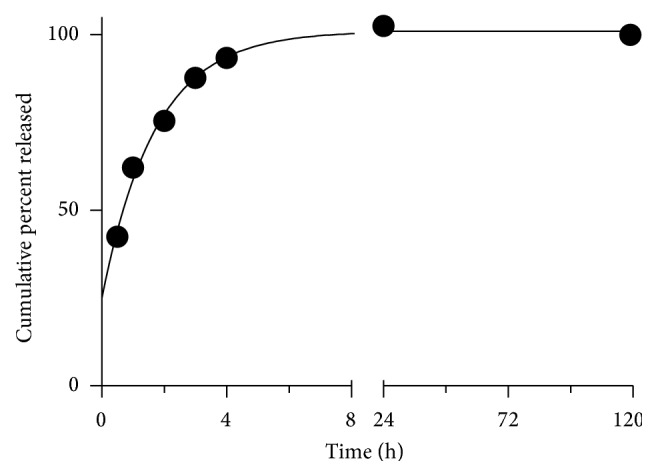
Release kinetics of PLGA-CS-NVP NPs in pH 7.2 at 37°C. Data are fit using the first-order model.

ARV release in various storage conditions was determined (Figures 4–7). At 4°C, there was no significant initial burst release of LMV. For both ARVs, consistent but lower drug amounts were released (Figure 5) compared to that at 37°C. At 4°C, water absorption and hydration of PLGA and/or chitosan within the NP may have decreased. This resulted in reduced NP degradation and subsequently less LMV being released.

During NP fabrication, ARVs may be adsorbed to the NP surface, entrapped within the NP, or chemically conjugated to the NP [41], or the NP may be a nanocrystal of the ARV itself [42]. Release of ARVs from PLGA NPs is initially controlled by diffusion, whereas in the final stages ARV release is controlled by dissolution/erosion [43, 44]. The release of NVP is primarily dependent on the degradation of the PLGA core and NVP's poor water solubility. Additionally, ionic interactions between NVP and PLGA may result in shielding PLGA's terminal carboxyl group. This shielding reduces the hydrophilicity of the polymer, thereby lessening the PLGA erosion and its concomitant NVP release [45]. The observed initial burst release of LMV suggests that once the hydrophobic PLGA core is exposed and perhaps partially disrupted, a portion of the LMV is immediately physically released and quickly diffuses through the outer, hydrophilic CS shell, although some LMV may eventually be adsorbed on the outer surface [46]. The slower, later release of LMV could result from the rest of the LMV that is entrapped within the PLGA core being more slowly released as the PLGA core undergoes less rapid dissolution.

Based on the best fit regression model, NVP exhibited a first-order release mechanism in pH 7.2 (Figure 7). The release of LMV in pH 5 was determined to be zero order (Figure 6). At this pH, late lysosomes and endosomes play an important role in the degradation of foreign matter in the endocytotic pathway [38]. In the presence of lysosomotropic agents in HIV patients, the release profile of LMV may play an important role in derailing HIV infectivity.

## 4. Conclusion

We have successfully fabricated positively charged, biodegradable PLGA-CS NPs that integrate both a hydrophobic and a hydrophilic ARV using a reproducible method. ARV release from a dual loaded NP, where one drug is released as a burst before the other, can be of therapeutic significance in HIV management. The initial LMV burst followed by a decrease that is paralleled by an increase in NVP observed in PBS pH 7.2 at 37°C may entail efficacy in inhibiting enzymes targeted by these ARVs at different times. This phenomenon may go a long way in managing viral load in the plasma and possibly prolong the ability of the HIV virus to develop resistance. The stability of these core-shell NPs demonstrates the ability to reformulate two first-generation ARVs into a nanoformulation with more favorable properties. In resource-limited settings, the stability of ARV-loaded NPs at room temperature would reduce expenses associated with cold-chain logistics and distribution, allowing for increased access to these medications. The observed stability of nonlyophilized NPs with 10% sucrose may be another storage strategy where lyophilization equipment may not be readily available. Our approach to fabricating dual loaded NPs, with stable physicochemical properties, offers the potential of further developing novel nanoformulations for use in HIV-1 infection. Further investigation of these core-shell ARV nanomedicines, using cellular and animal models of HIV-1 infection, will provide information critical to their clinical advancement.

## Figures and Tables

**Table 1:** Average size, PDI, zeta potential, and encapsulation efficiency of single and dual drug loaded NPs.

Sample	Components	Size (nm)	Polydispersity index (PDI)	Zeta potential (mV)
1.1	PLGA-CS-LMV	233	0.084	+25
1.2	PLGA-CS-LMV	231	0.072	+19
1.3	PLGA-CS-LMV	240	0.127	+28
1.4	PLGA-CS-LMV	244	0.148	+25
Mean		237	0.108	+24
Std. Dev.		6	0.04	3.9
Average EE%		7.5%		

2.1	PLGA-CS-NVP	317	0.314	+18
2.2	PLGA-CS-NVP	250	0.245	+22
2.3	PLGA-CS-NVP	293	0.272	+20
2.4	PLGA-CS-NVP	273	0.206	+22
Mean		283	0.259	+20.4
Std. Dev.		29	0.05	2.0
Average EE%		7.5%		

3.1	PLGA-CS-NVP-LMV	246	0.176	+23
3.2	PLGA-CS-NVP-LMV	242	0.131	+25
3.3	PLGA-CS-NVP-LMV	261	0.151	+15
3.4	PLGA-CS-NVP-LMV	220	0.122	+21
Mean		242	0.145	+21
Std. Dev.		17	0.02	4.2
Average EE%		13%		

4.1	PLGA-CS	227	0.010	+21
4.2	PLGA-CS	242	0.118	+18
Mean		235	0.064	+20
Std. Dev.		11	0.08	1.8

5.1	PLGA-LMV	278	0.125	−21

6.1	PLGA-NVP	306	0.305	−9

**Table 2:** Comparison of NPs size determinations by DLS and NTA.

Formulation	Dynamic light scattering (DLS)		Nanoparticle size tracking analysis (NTA)
	By intensity	By number	By number
PLGA-LMV	279	169	169
PLGA-CS-LMV	234	130	170
PLGA-CS	227	147	156
